# Medicolegal Issues in Hair Transplantation: A Review of North American Court Decisions and Canadian Regulatory Complaints

**DOI:** 10.1111/jocd.71132

**Published:** 2026-08-23

**Authors:** Aditya K. Gupta, Elizabeth Teasell, Mesbah Talukder

**Affiliations:** ^1^ Division of Dermatology, Department of Medicine, Temerty Faculty of Medicine University of Toronto Toronto Ontario Canada; ^2^ Mediprobe Research Inc. London Ontario Canada; ^3^ School of Pharmacy BRAC University Dhaka Bangladesh

**Keywords:** complaints, hair restoration, hair transplant, litigation, medicolegal, surgery

## Abstract

**Background:**

Hair transplantation is a common elective cosmetic procedure, and review of associated medicolegal claims may identify recurrent sources of patient dissatisfaction.

**Objective:**

To describe the characteristics, underlying allegations, and outcomes of hair transplant‐related litigation in North America and regulatory complaints in Canada.

**Methods:**

A retrospective review of Lexis Advance Quicklaw and Westlaw Edge legal databases was conducted from 1980 to 2026 to identify North American cases involving hair transplantation. Eligible cases included patient‐initiated claims against surgeons or clinics. Canadian regulatory complaints were identified through CanLII (Canadian Legal Information Institute).

**Results:**

Thirty‐five legal cases were included. The most common legal claims were medical malpractice and lack of informed consent. Reported outcomes included dissatisfaction with cosmetic results (10/35) and clinical adverse outcomes (25/35), the most common being scarring (13/35). Allegations frequently involved inadequate informed consent regarding risks, procedural details, and alternatives, as well as failure to deliver promised results (13/35), often linked to false or misleading representations by surgeons (11/35) or advertising materials (5/35). Six Canadian regulatory complaints were identified.

**Conclusion:**

Hair transplant litigation and regulatory complaints commonly arise from unmet expectations, alleged misrepresentation, and consent‐related deficiencies. Clear communication regarding risks, surgical outcomes, and surgical roles may reduce medicolegal risk.

## 1 Introduction

Hair transplantation is a commonly performed elective aesthetic surgical procedure worldwide. The 2022 Practice Census by the International Society of Hair Restoration Surgery estimated that approximately 149 254 surgical hair restoration procedures were performed in the United States and Canada in the preceding year, the majority of whom were men [1]. Understanding the medicolegal issues associated with these procedures is increasingly important for informing best clinical practice and strengthening patient counseling, informed consent, and safety‐focused regulatory and educational efforts.

Elective aesthetic procedures present unique medicolegal challenges because they are primarily undertaken to improve appearance rather than treat life‐threatening disease, and patients often pursue surgery with highly specific expectations regarding cosmetic outcomes [2]. Consequently, dissatisfaction with results or perceived discrepancies between expected and achieved outcomes may contribute to legal claims [3]. Inadequate informed consent, poor preoperative and postoperative care, and failure to establish realistic patient expectations have all been recognized as factors contributing to litigation against healthcare professionals performing aesthetic surgeries [4, 5]. A previous study examining hair transplantation malpractice through Westlaw jury verdicts and settlements reported that litigation most commonly involved allegations of inadequate informed consent and scarring, with the majority of cases decided in favor of defendants [3].

In the previous review, many legal decisions, including appeals, procedural rulings, and default judgments, were not examined. By systematically reviewing these additional sources of North American disputes from Lexis Advance Quicklaw and Westlaw Edge legal databases, alongside Canadian regulatory complaints from CanLII (Canadian Legal Information Institute) database, the present study provides a broader characterization of the legal claims, underlying allegations, and recurring factors contributing to medicolegal disputes in hair transplantation.

## 2 Methods

A retrospective review of publicly available court proceedings involving hair transplantation practice, including medical malpractice and negligence claims, was conducted using the Lexis Advance Quicklaw and Westlaw Edge (Thomson Reuters) legal databases from 1980 to June 4, 2026. These research platforms index federal, state/provincial, and appellate court decisions from the United States and Canada. Search terms related to hair transplantation (“hair transplant,” “hair graft,” “hair plugs,” and “hair restoration”) were combined using the Boolean operator OR. Retrieved records were screened for relevance, and duplicate lawsuits were removed (Figure S1). Cases were screened by one reviewer (E.T.) and in the case of uncertainty, a second author (A.K.G.) was consulted. As only publicly available legal records were searched, confidential settlements, mediation, arbitration, pre‐litigation claims, and unpublished disputes were not captured.

### 2.1 Inclusion and Exclusion Criteria

Cases involving hair transplantation or hair restoration procedures were included if they contained information on the underlying allegations or legal causes of action, regardless of case stage. Trial decisions, settlements, default judgments, procedural rulings, and appellate decisions were eligible when they provided sufficient information regarding the underlying dispute. When multiple records existed for the same case, the case was included only once, with all available documents reviewed to capture the most complete information. Cases addressing only procedural or jurisdictional issues without substantive information on the underlying allegations were excluded, as were cases that did not involve claims against a physician or hair restoration clinic/corporation providing hair transplantation services or where the alleged injury was incidental to, rather than arising from, the hair transplantation procedure or its perioperative management, cosmetic outcome, informed consent, or other aspects of hair transplant care. Privacy/publicity claims when they pertained directly to hair transplant procedure photos or videos were included.

To identify Canadian tribunal and disciplinary decisions, the same search strategy was applied to the CanLII database, which provides public access to decisions from Canadian courts and administrative tribunals, including disciplinary and review bodies. Relevant decisions from the Health Professions Appeal and Review Board involving physician complaints related to hair transplantation were included. As this study consisted solely of a review of publicly available legal records and tribunal decisions, institutional research ethics board approval was not required. Tribunal decisions regarding physician complaints were not systematically reviewed for the United States as there are no single publicly available databases for these decisions, which differ across states and are not systematically searchable within state databases.

## 3 Results

### 3.1 Hair Transplant Litigation

Of the publicly reported legal disputes retrieved from the Lexis Advance Quicklaw and Westlaw Edge databases, 32 cases from the United States and 3 from Canada were eligible for inclusion (Figure S1). A summary of all the included cases can be found in Table S1. The majority of cases in the United States occurred in the Northeast United States (15/32, 47%), especially New York, the Southeast (7/32, 22%) and the West (6/32, 19%), namely California. Most cases were adjudicated between 2000 and 2010 (17/35, 49%), with 11 cases prior to 2000 and 7 cases between 2010 and 2026. The included lawsuits named 35 unique American and Canadian surgeons and 19 unique corporations or hair transplant clinics as defendants. Some lawsuits involved multiple defendants, and some surgeons and corporations were named in more than one lawsuit. Hair restoration clinics and surgeons most often faced three major categories of liability and could be accused of multiple categories simultaneously: (1) medical malpractice/negligence, (2) lack of informed consent, (3) consumer fraud/false advertising, and (4) breach of contract. The specific legal claims and the underlying sources behind these claims are summarized in Table 1.

**TABLE 1:** Litigation characteristics.

Legal causes of action^a^	No. of cases (%)	Alleged breaches^a^	No. of cases (%)	Clinical/aesthetic outcomes^a^	No. of cases (%)
Medical malpractice	24/35 (69%)	Inadequate informed consent^a^	17/35 (48%)	Unnatural looking hair/hairline	5/35 (14%)
Lack of informed consent	17/35 (48%)	Risks/surgery outcome	9/35 (26%)	Dissatisfactory outcome	3/35 (9%)
Negligence	10/35 (29%)	Possible alternatives	3/35 (9%)	Lack of hair growth	2/35 (6%)
Breach of contract	9/35 (26%)	Procedural details	5/35 (14%)	Reported adverse outcomes^a^	25/35 (71%)
Fraud	7/35 (20%)	Qualifications/delegation	1/35 (3%)	Scarring	13/35 (37%)
Deceptive business practices^b^	6/35 (17%)	Failure to deliver on promises	13/35 (37%)	Pain	5/35 (14%)
Putative damages	5/35 (14%)	Cosmetic outcome	10/35 (29%)	Infection	4/35 (11%)
Negligent misrepresentation	3/35 (9%)	Number of grafts	3/35 (9%)	Numbness/tingling	4/35 (11%)
Privacy/confidentiality/publicity claims	3/35 (9%)	Misrepresentations (surgeon/staff)	11/35 (31%)	Excessive bleeding	3/35 (9%)
Assault/battery	2/35 (6%)	Poor surgical technique	11/35 (31%)	Aggressive hair loss	2/35 (6%)
Breach of express/implied warranty	2/35 (6%)	Misleading or false advertising^c^	5/35 (14%)	Scalp necrosis	2/35 (6%)
Loss of consortium	1/35 (6%)	Provide standard of care	5/35 (14%)	Head wound/laceration	2/35 (6%)
		More surgeries than indicated	5/35 (14%)	Hematoma	1/35 (3%)
		Provide standard of care	5/35 (14%)	Mild thrombophlebitis	1/35 (3%)
		Patient selection/pre‐surgical evaluation	3/35 (9%)	Stroke	1/35 (3%)
		Inadequate postoperative care	3/35 (9%)	Unsutured excision	1/35 (3%)
					
				**Liability‐context issues**	**No. of cases (%)**
				Improper delegation/physician absent	3/35 (9%)
				Use of patient photographs or video	3/35 (9%)
				Failure to supervise technicians	2/35 (6%)
				Poor documentation	1/35 (3%)

^a^ Categories are not mutually exclusive.

^b^ Consumer protection law, negligent misrepresentation, false advertising and deceptive acts in business.

^c^ Advertisements, promotions, commercials, brochures.

Judicial or final administrative outcomes were available for 23 cases (Table 2). Of these, 10 proceeded to jury trial, resulting in evenly split verdicts, with five cases decided in favor of the defendants and five in favor of the plaintiffs. Among these cases, medical complications relating to the surgical procedure were common (7/10). Reported complications included extensive scarring, persistent wounds, scalp lacerations, permanent hair loss, infection, pain, and postoperative swelling. Scarring was the most frequently reported complication in this subgroup (6/10), often described as wide or permanent visible scarring. One case involved a severe neurological complication in which the patient experienced a stroke leading to moderate brain injury, speech impairment, and hemiplegia. It was alleged that the surgeon failed to adequately perform the pre‐surgical evaluation and appropriately disclose the risks associated with the patient's age.

**TABLE 2:** Judicial outcome.

Judicial outcome	No. of cases (% of cases with reported outcomes)^a^
Plaintiff success	8/22 (36%)
Jury verdict for plaintiff	5
Default judgment for plaintiff	1
Judgment (court)	2
Defendant success	11/22 (50%)
Jury verdict for defendant	5
Summary judgment for defendant	3
Trial judgment	1
Lower court judgment (dismissal)	2
Procedural dismissal (e.g., time‐barred)^b^	3/22 (14%)
Mixed outcome pre‐trial	9/35 (26% of total cases)
Unknown	4/35 (11% of total cases)

^a^ Percentages are calculated based on the subset of cases with identifiable judicial or administrative outcomes for given outcomes.

^b^ Procedural dismissals included cases dismissed due to statute of limitations or default after failure to appear for jury selection.

Issues related to informed consent were also frequently identified in juried cases (7/10). These allegations typically involved inadequate disclosure of surgical risks and complications, including permanent tissue damage, uncertainty regarding the number of required procedures, and insufficient explanation of intraoperative processes.

Across the full cohort of cases (*n* = 35), medical adverse outcomes were frequently cited in litigation (25/35, 71%). Common adverse outcomes included scarring, numbness, infection, bleeding, and pain (Table 1). Additionally, aesthetic concerns were frequently reported, including unnatural‐appearing results such as a “pluggy” or doll‐like appearance or an artificial‐looking hairline, overall dissatisfaction with surgical outcomes, and perceived lack of hair growth following the procedure (10/35, 29%). Most cases involved mild adverse outcomes, often accompanied by allegations of inadequate informed consent regarding the possibility or likelihood of risks, particularly the degree of scarring, as well as claims that specific promises or guarantees about the cosmetic result had not been fulfilled.

Deficiencies in informed consent were common, occurring in 17 of the 35 cases. In addition to failures to adequately disclose procedure‐specific risks, failure to inform of the specific details of the procedure was also noted. More specifically concerns also arose from the lack of communication regarding the extent of surgeon involvement and the roles performed by technicians or nurses during surgery. Additional consent‐related concerns included failure to discuss alternative treatment options, variability in outcomes based on donor hair characteristics (e.g., scalp laxity and donor site availability), and the potential need for multiple procedures to achieve desired cosmetic results.

The other major underlying source of allegations was failure to deliver on promised outcomes (13/35, 37%) (Table 1). These disputes frequently arose from patient expectations that plaintiffs alleged were shaped by false or misleading representations made prior to the procedure. Such representations were conveyed through both advertising materials (5/35, 14%) and statements attributed directly to surgeons or clinic staff (11/35, 31%), with some cases involving allegations related to both sources. Plaintiffs alleged that promotional materials, brochures, or verbal communications included assurances or guarantees that were not ultimately fulfilled, such as promises that “a single transplant will result in a full head of permanent hair,” “you will never go bald again,” the procedure used a “virtually painless technique,” or that transplanted hair was “guaranteed to grow for the rest of your life.”

Separate allegations involved the unauthorized use of patient photographs or videos for marketing purposes beyond the scope of consent, resulting in claims of invasion of privacy and breach of agreed‐upon restrictions on image use. Two privacy‐related cases resulted in settlements. In one case, a clinic used patient photographs outside the agreed geographic region and, despite assurances that the advertisements would cease, continued displaying the images. In another case, patient video footage was broadcast on BCTV despite the patient having consented only to educational use. Allegations of assault and battery were uncommon (2/35, 6%) and generally arose in the context of disputes regarding unauthorized or non‐consensual aspects of surgical conduct.

Overall, cases were often dismissed before making it to trial due to summary judgments for the defendant or procedural dismissals (Table 2), most often because cases were lodged beyond the time allotted for an eligible claim to be made. Dismissals or partial dismissals due to time‐barred cases often occurred with medical malpractice claims as they have a legal deadline for the lawsuit to be filed, typically around 1–3 years.

Hair transplant surgeries resulting in death have been reported exceedingly rarely in North America. In this review, only one death case was identified. It involved allegations of medication management failures and delayed emergency response during a hair transplant procedure in California. An additional recent case in Florida, outside the cases captured in the databases, involved fatal complications related to local anesthetic systemic toxicity (LAST) that occurred while the patient was being prepared for hair transplantation. The case was settled before formal litigation [6]. Although uncommon, these cases highlight the potential for catastrophic outcomes in elective cosmetic procedures and underscore that not all cases with serious adverse events may be captured with available legal databases.

### 3.2 Hair Transplant‐Related Canadian Regulatory Body Complaints

The CanLII database identified six relevant complaints (Figure S1) involving Canadian hair transplant surgeons that were reviewed by provincial Health Professions Review Boards (Table S2). Complaints submitted to the provincial Colleges of Physicians and Surgeons broadly fell into two themes. First, patients alleged that they had been misled about aspects of the procedure, including the role of technicians and nurses. Second, patient concerns indicated the expected quality of operation was not provided. Overall complaints generally included some nature of dissatisfaction regarding the outcome of the surgery, including the surgery not being performed as the patient had requested (Table 3). The most frequently reported specific complaint (4/6, 67%) was that the surgeon was not present during substantial portions of the procedure and technicians performed procedure unsupervised.

**TABLE 3:** Summary of nature of complaints against hair transplant surgeons lodged with Canadian College of Physicians and Surgeons.

Complaint	No. of cases (%)
Dissatisfied with surgical outcomes	6/6 (100%)
Surgeon not performing procedure/unsupervised technician involvement	4/6 (67%)
Inadequate follow‐up	3/6 (50%)
Inadequate/inconsistent documentation	3/6 (50%)
Emotional distress due to surgery	2/6 (33%)
Scarring	1/6 (17%)
Informed consent concerns^a^	2/6 (33%)
Grafts lower than agreed upon	1/6 (17%)
Other^b^	2/6 (33%)

^a^ Regarding failure to discuss alternative surgical options, or risks associated with surgical choice, or video recording.

^b^ Including surgeon accused of being on drugs and surgeon accused of discrimination.

## 4 Discussion

This study expands upon previous work by examining a broad spectrum of publicly available medicolegal records. By including court decisions across multiple stages of litigation, as well as Canadian regulatory complaints, we have extensively characterized the underlying sources of allegations and recurring factors contributing to disputes against hair transplant surgeons and clinics. The publicly available cases identified in this review suggest that medicolegal disputes more often involved deficiencies in communication, informed consent, expectation setting, transparency regarding surgeon involvement, and advertising practices than technical surgical negligence. Although complaints and allegations of complications and adverse effects were common, they most often reflected dissatisfaction with aesthetic outcomes, with only rare instances of severe medical events.

Medicolegal action in plastic surgery is known for having specific common sources underlying the claims [2, 7]. Based on a retrospective analysis of Canadian Medical Protective Assocaition data, the annual incidence of regulatory body complaints and civil‐legal actions for plastic surgeon members between 2013 to 2017 was estimated at 12.1% and 6.7%, respectively [7]. This agrees with estimates from the United States indicating plastic surgeons having a 15% likelihood of being sued annually [8]. While few of these cases go to trial and overall 75% of the cases end in no payment [8], there is still a prerogative to reduce potential for lawsuits and complaints. That being said, compared to some plastic surgeries, such as abdominoplasty and blepharoplasty, which are performed at similar frequencies to hair restoration procedures, the frequency of published litigation cases in hair restoration litigation is overall lower [9, 10].

The patient experience in hair transplantation often begins before the patient enters the clinic, through exposure to advertising and promotional materials, and continues with preoperative consultations involving the surgeon and clinical staff. At both stages, the surgeon and clinic have a responsibility to ensure that procedural details, anticipated outcomes, and treatment limitations are represented accurately. This includes counseling patients regarding realistic expectations for hairline design, graft density and coverage, the potential need for staged or additional procedures, and the inherent variability of cosmetic outcomes. Where possible, guaranteeing specific aesthetic outcomes should be avoided; instead, outcomes should be represented as estimates rather than assurances [11], with careful documentation of these discussions serving to further reduce medicolegal risk. These principles are reflected in the American Board of Hair Restoration Surgery (ABHRS) Code of Ethics and Truth in Representation and Advertising Guidelines, which require truthful and non‐misleading advertising, accurate representation of credentials, and avoidance of promotional claims such as guaranteed results, “scarless” surgery, hair multiplication, risk‐free procedures, or claims that additional procedures will be unnecessary [12].

Although relatively uncommon, several reviewed cases highlighted deficiencies in preoperative evaluation and patient selection. Comprehensive preoperative assessment should identify factors that may increase the risk of adverse events, suboptimal aesthetic outcomes, or patient dissatisfaction. Consideration of the underlying cause and extent of hair loss, donor hair availability, scalp laxity, patient age, medical comorbidities, and patient expectations may help determine procedural suitability and identify individuals who may benefit from alternative treatment approaches or more extensive preoperative counseling. The discussions initiated during advertising, preoperative counseling, and patient selection should culminate in a thorough informed consent process. Comprehensive informed consent is essential to reduce misunderstandings, facilitate shared decision‐making, and improve patient satisfaction. Lack of informed consent is a common allegation in medicolegal claims, including those involving aesthetic procedures [13]. In a retrospective review of lawsuits pertaining to five types of plastic surgery (e.g., abdominoplasty, breast augmentation, breast reduction, blepharoplasty, facelift), a lack of informed consent was associated with a higher likelihood for a non‐defense verdict compared to cases without consent issues [9]. Accordingly, consent should involve clear upfront communications regarding the nature of the procedure, the anticipated benefits and limitations, disclosure of team roles, possible alternative therapies, patient specific risks, and the use of patient photos and videos [2, 14].

Notably, a large portion of patients in the current review reported a lack of consent regarding procedural risks, including both medical complications, and the possibility of undesirable aesthetic outcomes, as well as details of the procedure itself, physician availability during surgery, and the delegation of key procedural tasks to nurses and technicians. These concerns highlight the importance of disclosure of team roles, including responsibilities of all individuals involved in the procedure. Inadequate physician supervision and inappropriate delegation of surgical tasks to nurses and technicians were repeatedly identified in physician complaints and several legal cases. Clinician‐related factors and failures in communication or coordination within the healthcare team were identified in 75% of cases from a review of Canadian Medical Protective Association medicolegal cases involving hair loss treatment [15]. Ensuring appropriate physician oversight, clearly defining the scope of delegated activities, and documenting team involvement may help prevent misunderstandings regarding care delivery.

In addition to transparency‐related issues, adverse outcomes also contributed to litigation and complaints. Scarring was the most commonly alleged physical injury in litigated cases, consistent with findings from broader cosmetic surgery malpractice literature where dissatisfaction or scarring was described in 43% of cases [9]. Following scarring, pain and sensory disturbances were the most common adverse events reported in litigation cases and regulatory complaints; pain and discomfort are estimated to occur in approximately 66% of hair transplant complications reported in non‐case studies [16]. Necrosis was an uncommon cause of litigation, with only one case identified in our review. Although recipient‐site necrosis is rare, occurring in an estimated 0.03% of patients [17], its severity may contribute to a disproportionate medicolegal impact when it does occur. While complications cannot be eliminated, comprehensive preoperative patient evaluation, appropriate counseling regarding potential risks, effective communication, and meticulous postoperative management may reduce the likelihood of severe adverse outcomes. Ongoing postoperative communication, appropriate management of complications, and documentation of follow‐up assessments and patient concerns are essential to support continuity of care and reduce medicolegal risk.

This study has several limitations inherent to its sources. Searches were restricted to publicly available court and tribunal decisions in Lexis Advance Quicklaw, Westlaw Edge, and Canadian tribunal decisions via CanLII. Consequently, the analysis does not capture the majority of disputes resolved through confidential settlements, mediation, arbitration, or pre‐suit negotiations, practices especially common in high‐stakes cases involving severe injury or death. It, therefore, likely underestimates the scope and frequency of disputes arising from hair transplant procedures. United States physician regulatory and disciplinary actions were not systematically reviewed as they differ by state and physician licensing board databases generally do not permit systematic searching by procedure or allegation, creating an asymmetry with the Canadian data and an underrepresentation of United States Medical board action. Additionally, regulatory complaint data were limited to cases reviewed by the Health Professions Review Board and may not represent all complaints filed. Reported cases frequently lacked complete clinical details and final outcomes of the litigation. Recent cases (post‐2010) may be underrepresented, due to ongoing proceedings or private resolutions. Collectively, these constraints likely underestimate the true frequency and full spectrum of medicolegal issues in hair transplant.

## 5 Conclusion

The findings of this review suggest that, among publicly available hair transplantation medicolegal cases in North America, disputes most commonly arise from issues related to patient–surgeon communication and expectation management rather than primarily from technical errors. Although the identified cases represent only publicly accessible legal records and likely underestimate the full spectrum of disputes, they highlight recurring areas of concern that may inform risk mitigation strategies. Comprehensive patient counseling, standardized informed consent, transparent advertising practices, appropriate supervision and disclosure of delegated responsibilities, careful patient selection, and meticulous documentation may all contribute to reducing disputes while promoting safer, more patient‐centered care. In addition to individual clinician efforts in communication and documentation, the specialty may benefit from enhanced systems for transparent reporting and learning from rare but serious adverse outcomes. Greater attention to these domains may help better align patient expectations with achievable outcomes and mitigate medicolegal concerns.

## Author Contributions

Conception of the manuscript was done by A.K.G. The manuscript was drafted by E.T. and A.K.G. The manuscript was substantively edited and revised by A.K.G., E.T., and M.T.

## Conflicts of Interest

The authors declare no conflicts of interest.

## Supporting information


**Figure S1:** Databases search workflow for North American litigation cases (A), and Canadian regulatory complaints (B).

**Table S1:** North American litigation cases concerning hair transplant procedures.
**Table S2:** Canadian regulatory complaints concerning hair transplant procedures.

## Data Availability

Data sharing not applicable to this article as no datasets were generated or analysed during the current study.
