# Supplementary material for: Medicolegal Issues in Hair Transplantation: A Review of North American Court Decisions and Canadian Regulatory Complaints
Source: J Cosmet Dermatol. 2026 Aug 23;25(8):e71132. doi: 10.1111/jocd.71132 (PMC13500931; doi:10.1111/jocd.71132)
Supplement: Supplementary file 1 [file JOCD-25-e71132-s001.docx]

Supplementary table 1. North American litigation cases concerning hair transplant procedures

| **Case name** | **Year of incident**  **Year decision** | **Jurisdiction** | **Defendant type** | **Hair transplant technique**  **(if available)** | **Main legal causes of action** | **Core dispute** | **Clinical outcome** | **Judicial outcome** |
| --- | --- | --- | --- | --- | --- | --- | --- | --- |
| *St. John v. Bosley Inc* | 2008-2009  2021 | Ohio | Corporation, surgeon | FUE | Bodily injury, Breach of contract,  Race discrimination | Failure to remove degree of scarring promised (told he could remove up to 85% but only 50% was removed), Terrible transplant | Bleeding and scabbing, unsutured excision in head, nerve damage resulting in tingling or twitching | Summary judgement – procedural dismissals |
| *Turner v. Bosley Medical Institute* | 2016  2019 | Louisiana | Medical group, surgeon | FUT | Medical malpractice | Failure to properly sanitize and close wound site resulting in infection, failure to treat infected wound | Infection | Summary judgement |
| *McCabe v. Panine* | 2012  2019 | Illinois | Surgeon, Medical group | FUT | Medical malpractice, lack of informed consent | Negligent performed surgery and failed to inform of risks (e.g. scarring, permanent skin damage)  Improper use of silver nitrate and cortisone  Failure to address complaints | Scarring greater than 3cm | Jury verdict for defense |
| *Betoushana v. Meshkin* | 2017 | California | Medical group, Corporation, surgeon | n/a | Violations of civil code, 1770 (deceptive practices), negligence, breach of contract, putative damages | Failure to provide number of grafts agreed upon | Number of grafts lower than desired | Sustained in part, dismissed in part |
| *Colson v. Samson Hair Restoration* | 2009  2013 | North Carolina | Medical group/ corporation, surgeon | n/a | Professional negligence,  Common law negligence  Loss of consortium | Damages arising from hair transplant procedure including scalp necrosis | Scalp necrosis in hair transplant area | Default judgement with monetary damages: $1,077,765.42 |
| *Schecter v. Bosley Med. Group, P.C* | 2008  2013 | New York | Medical group, surgeon | FUT | Medical malpractice, Negligence, lack of informed consent, violations of General Business Law 350 (false advertising) | Surgeon removed excessive skin resulting in pulling, lack of documentations of pre-operative scalp examination and, procedural details, consent did not warn about necrosis, did not discuss alternatives | Excessive bleeding and pain, wide scar, shock loss in donor area which expert witness alleged was actually necrosis | Medical malpractice and lack of informed consent sustained to trial, summary judgement denied |
| *Salmanzadeh v. Bosley Med Group* | 2010  2012 | California | Surgeon, medical group, corporation | n/a | Medical malpractice, lack of Informed consent | Failure to perform adequate pre-surgical evaluation, failure to informed of age-associated risks of procedure, consent on that performing anesthesia and monitoring during surgery, failure to detect mid-procedure stroke, failure to refer post-surgery | Brain damage: hemiplegia, stroke, speech impairment | Jury in favor of plaintiff Damages awarded: $1,238,534 |
| *Strong v. Cosmetic Restoration Mktg.* | 2002-2006  2009 | New York | Corporation | FUT | Medical malpractice, breach of contract | Poor recovery from transplant procedure | Wound on the back of head did not heal properly | Procedural dismissal (time barred) |
| *Cardaci v. Ciarello* | 2002  2007 | New York | Surgeon | Micrograft technique hair transplantation | Medical negligence, lack of informed consent | Failed to perform procedures properly, failed to take precautions to avoid vascular injury and failed to inform of unwarranted complications | Mild thrombophlebitis (swelling of the vein) | Trial court orders – summary judgement for defendant |
| *Pettineo v. Sceintific Image Ctr Staffing* | 2007 | Michigan | Hair restoration surgeon | Autologous graft hair transplantation | Medical malpractice  Negligent Hiring & supervision  Vicarious Liability | Failure to exercise skill and caution in transplantation and to diagnose and treat wound infection  Failure to supervise and train their employees | Scar compression with hyperpigmentation | Jury verdict for defense |
| *Ago v. Pandeolfino Hair Corp.* | 1995  2007 | Massachusetts | Hair restoration group, hair restoration surgeon | FUT | Medical malpractice Breach of express and implied warranty Fraud & misrepresentation | Failure to fully inform patient about procedure, including nature and scope of procedure, level and degree of skill or surgeon, probability of success, risks of procedure, and to properly perform transplant and breach of warranties | Infection  Improperly planned and grafts misplaced | Plaintiff verdict settlement of 20000 |
| *Ruffino v. Neiman* | 2005 | New York | Dermatologist | n/a | Breach of physician-patient confidentiality, violation of Civil rights law sections 50-51 (unauthorized use of likeness), punitive damages | Unauthorized publication of before-and-after photographs in advertising | None | Summary judgement for defendant |
| *Zieve v. Hairston* | 2004 | Georgia | Surgeon and corporation | n/a | Tort Breach of contract | Invasion of privacy based on public disclosure, Fraud claim based upon representation in two phone calls | None | Jury verdict for plaintiff Damages: $365,000 |
| *Bayer v. Bosley Medical Group, Dr. Aragoni* | 2004 | California | Medical Group, surgeon | FUT | Medical malpractice, medical battery | Secondary procedure was performed to obtain more grafts as the original incision did not yield the number of grafts desired | Scarring | Dismissed on summary judgement |
| *Gottlieb v. Handler* | 1992-1996  2003 | New York | Medical group, surgeons |  | Medical malpractice, lack of informed consent, common law fraud | False/misleading advertising and representations made by physician, failure to achieve desired outcome, continued need for additional procedures | Dissatisfactory result | Partial summary judgment |
| *Romain v. Macvaugh, Strenio, Certo* | 1993-1995  2003 | Pennsylvania | Surgeons | n/a | Negligence, lack of informed consent | Failure to screen properly for surgical candidacy, misrepresentations by staff, technician developed treatment plan not physicians, failure to perform procedures properly | “Pluggy” baby doll like hair, scarring, | Partial summary judgement for some physicians |
| *Smith v. Cattani* | 2003 | New York | Plastic surgeon | FUT? | Medical malpractice, lack of informed consent | Inappropriate patient selection based on abnormal pattern of hair loss, failure to diagnose of investigate underlying cause of hair loss/ seek dermatologist consultation, poor donor site selection, technical planning affecting long-term graft survival | n/a | Partial reversal of summary judgement (medical malpractice sustained, informed consent dismissed) |
| *Bollion v. Hitzig* | 1994-1995  2001 | New York | Medical group, Surgeons |  | Medical malpractice, lack of informed consent  Common law fraud, Violations of General Business Law 349, 350,  Breach of contract Punitive damages | Failure to deliver on claims promised, more surgeries than promised, deceptive or misleading advertising | Transplant resulted in gaps, scars and plugs, numbness in ears, intermittent bleeding in scalp and pain, scarring fake looking hairline | Dismissed in part, sustained in part |
| *Abrams v. Handler* | 2001 | New York | Surgeon |  | Medical malpractice, breach of contract, negligent misrepresentation, violations of General business law 349 and 350, punitive damages | Outcome of procedure did not meet promises, surgeon was absent for the majority of the surgery | Unsatisfactory outcome | Dismissed in part  Sustained in part |
| *Bellera v. Handler* | n/a  2001 | New York | Surgeons | n/a | Negligent misrepresentation, medical malpractice, common-law fraud, lack of informed consent | *joint case 21 plaintiffs accused. Promised that 1-4 procedures would permanently restore hair with no ongoing maintenance required. | Unnatural hairlines, scarring, numbness, pain | Dismissed all but one patient (dismissed plaintiffs could refile their lawsuits)  Medical malpractice claims to continue  Final outcome unknown |
| *Soskel v. Handler* | 1991-1992  2001 | New York | Surgeon, Medical group | n/a | Breach of contract, violations of General Business Law 349 and 350 | Deceptive marketing practices, poor outcome of hair transplant | Scarring and unnatural ‘corn-stalk’ results | Dismissal- procedural/ legal doctrine (barred by res judicata/ time barred) |
| *Youmtoub v. Rosanelli* | 1998 | California | Hair restoration surgeon | Scalp reduction procedure | Medical malpractice | Negligent to properly perform procedure | Surgical scarring | Jury verdict for defense |
| *Boggs v. Bosley Med Inst.* | 1997 | Georgia | Surgeon |  | Medical malpractice, fraud | Continual misrepresentation of number of procedures needed | Significant scalp scarring, hair density lower than promised, continued need for additional surgeries | Dismissed in part (procedural), Fraud claim sustained, final outcome unknown |
| *Shombing v. Bosley Medical Group* | 1994  1997 | California | Medical group | n/a | Medical malpractice, lack of informed consent | Failure to properly inform of risks and complications of procedure and provide standard of care | n/a | Jury in favor of plaintiff Damages awarded: $134, 700 |
| *Romatowski v. Hitzig* | 1993  1996 | New York | Surgeon | Scalp reduction, followed by hair graft transplant | Medical malpractice, Lack of informed consent, Negligence, Breach of contract, Fraud, Assault and battery | Claimed specific promises were made regarding the outcome: scalp reduction and 50 grafts would completely cover baldness | Dissatisfactory results | **Dismissed on summary judgement**: 1) medical malpractice  2) lack of informed consent  3) fraud  4) assault and battery  **Allowed to proceed to jury trial**:  1)breach of contract |
| *Haase v. Starnes* | 1996 | Arkansas | Physician | Scalp reduction, hair transplant and grafts | Negligence, Breach of express warranty | Advertisements stating “We guarantee you a full, growing head of hair for the rest of your life” etc. during course of scalp reduction, hair grafts and transplants a permanent scar formed, breached the representations and warranties | Scalp infection which resulted in permanent scarring that prevented additional hair transplants | Judgement that expert testimony was necessary, outcome of case unknown |
| *Sardino v. Weidig* | 1993 | Maryland | Surgeon | Scalp reduction surgery | Lack of informed consent | Failure to inform of procedure until he was administering anesthesia | Scarring | Jury verdict for plaintiff Damages: $175,000 |
| *Georgiadis v. Bronson* | 1987  1992 | California | Surgeon | Scalp reduction and hair transplantation | Medical malpractice | Mispresenting on number of surgeries, limited qualifications of defendant, failure to meet standard of care | Scarring | Jury verdict for defense |
| *Scnick v. Wolf* | 1990 | Texas | Surgeon | n/a | Medical malpractice, Informed consent | Failure to obtain informed consent and negligent performed surgery | Head laceration, scalp infection | Jury verdict for defense |
| *Dixon v. Peters* | 1976-1978  1983 | North Carolina | Surgeon | Hair “plug” transplant, Juri-flap procedure | Medical malpractice, lack of informed consent | Negligence in performing procedures, failure to inform of risks and the possibility of visible scarring | No hair growth on transferred scalp, permanent scarring in visible areas on forehead | Dismissed |
| *Kutzgar v. Yarborough* | 1980 | Louisiana | Surgeon | Hair “plug” transplant | Medical malpractice, lack of informed consent | Lack of informed consent to cutting of the scalp, misleading pamphlets on hair transplant procedure, pain experienced with procedure | Lack of hair growth in plugs, pain, nerve damage resulting in 5” ovoid numbness in transplant area | Defendant dismissal |
| Canada | | | | | | | | |
| *Razban v. Peoples Hair Scalp* | 2007 | Ontario | Medical group |  | Pre-contractual representations, Negligent misrepresentation | Guarantees made in the contract were not meet, omitted information that grafts in plan would not provide desired outcome | Not full head of hair implied in contract  Pain | Plaintiff success- damages and costs $5, 675 |
| *Grimard v. Gilbert* | 2001 | Ontario | Surgeon |  | Medical malpractice, negligence | Representations regarding the transplant outcomes were not met following surgery | Unsatisfactory outcome | Unknown |
| *Hollinsworth v. BCTV Group Ltd.* | 1998 | British Colombia | Corporation |  | Invasion of privacy, unwanted publicity | Videos broadcasted on television without consent | None | Plaintiff awarded $15, 000 |

Supplementary table 2. Canadian regulatory complaints concerning hair transplant procedures

| **Case name** | **Year of incident**  **Year of decision** | **Location** | **Hair transplant technique**  **(if available)** | **Core complaint** | **Outcome** |
| --- | --- | --- | --- | --- | --- |
| J.S. v. R.G.J | 2013  2019 | Toronto | FUT | 1.Physcian did not perform the hair transplant himself - it was performed by nurse,  2.Did not implant agreed upon grafts,  3. No follow-up after procedure,  3.Transplants performed have caused great emotional distress, and impacted professional life | No action |
| N.M. v A.A | 2006  2009 | Toronto | Scar revision | 1. Failed to perform satisfactory hair transplant,  2. Technician performed procedure unsupervised, follow-up appointment preoperative photos were not available, and photos after were not available to the applicant, consent forms have dissimilar signatures, medical documentation is illegible and inadequate | Written caution - ensure consent documentation reflects that (1) he may not personally perform procedure- if that is the case, (2) documentation is organized and complete |
| Complaint v. College Physicians and Surgeons of British Columbia | 2022  2025 | British Columbia | FUE | 1. Disappointed with the results,  2. Left a scar,  3. Technician doing the surgery unsupervised (performing unsupervised incisions) | No action |
| S.V.K. v C.P.C | 2018  2020 | Ontario | FUE | 1. Failed to perform procedure requested,  2. Confusion over finasteride medication provided,  3. Performed procedure while on drugs,  3. Failed to maintain medical records,  4. Failed to provide copy of records to applicant when requested | No action |
| P.F. v C.T. | 2018  2020 | Ontario | FUE | 1. Standards falling below the reasonable standard of care,  2.. Physician not present in room during procedure, did not perform procedure after telling applicant he would,  3.. Videotaped procedure without consent,  4. Did not provide applicant with footage when requested | No action but noted physician is expected to ensure detailed communications with patients, and documentation of nature of planned procedures and anticipated results and follow through on promises made to patients |
| J.B. v R.G.J | 2018  2020 | Ontario | FUE | 1. Took hairs from the side of head without consent,  2. Placement of thick and thin hairs,  3. Perfectly aligned hairs so he has doll hair now,  4. Deliberately performed transplant to ensure poor surgical outcome because of religion and ethnicity | No action |
