# Supplementary material for: Medicolegal Issues in Hair Transplantation: A Review of North American Court Decisions and Canadian Regulatory Complaints
Source: J Cosmet Dermatol. 2026 Aug 23;25(8):e71132. doi: 10.1111/jocd.71132 (PMC13500931; doi:10.1111/jocd.71132)
Supplement: Supplementary file 2 [file JOCD-25-e71132-s002.docx]

**A**

Westlaw Edge	(Thomson Reuters)

Lexis Advance Quicklaw

United States (n=188)

Canada (n=36)

United States (n=72)

Canada (n=14) After selecting for relevant subjects (civil practice and procedure, health law)

Trial court orders (n=20)

Cases (n=20)

Jury verdicts + settlements (n=32)

Not related to hair transplant procedure (n=33)

Duplicates (n=9) Not designated for publication (n=1) Not related to hair transplant procedure (n=10)

Duplicates (n=2)

Not related to hair transplant procedure (n=12)

Duplicates/proceedings from same case (n=2) Missing case details concerning underlying allegation/ not against surgeon or clinic (n=5)

Not related to hair transplant procedure (n=164)

Duplicates (n=3) Missing case details (n=1)	Allegations incidental to hair transplant (n=1)	Not related to hair transplant (n=18)

Duplicates/ proceedings from same case (n=6)

Cases identified through references in analyzed cases (n=2)

Cases eligible for analysis (n=0)

Cases eligible for analysis (n=2)

Cases eligible for analysis (n=17)

Cases eligible for analysis (n=3)

Cases eligible for analysis (n=4)

Cases eligible for analysis (n=9)

Cases eligible for data collection (n=35)

**B**

Cases excluded (n=75)	1. Duplicates (n=1)	2. Not relevant to procedure (n=74)

Cases eligible for analysis (n=6)

Cases screened + assessed for eligibility (n=81)

Canadian Legal Information Institute (CanLII)

Supplementary Figure 1. Databases search workflow for North American litigation cases (A), and Canadian regulatory complaints (B).
